# Management of the Vertical Dimension in the Camouflage Treatment of an Adult Skeletal Class III Malocclusion

**DOI:** 10.1155/2020/8854588

**Published:** 2020-08-11

**Authors:** Manuel Gustavo Chávez Sevillano, Gina Judith Flores Diaz, Luciane Macedo de Menezes, Livia Kelly Ferraz Nunes, José Augusto Mendes Miguel, Cátia Cardoso Abdo Quintão

**Affiliations:** ^1^Department of Orthodontics, National University of San Marcos, Lima, Perú. Av. GermánAmézaga 375, Cercado de Lima-Lima, Peru; ^2^Orthodontics Professor of the School of Health and Life Science-Pontifícia Universidade Católica do Rio Grande do Sul (PUCRS), Brazil. Ipiranga Avenue, 6681 Partenon-Porto Alegre RS, Brazil; ^3^Department of Orthodontics, Rio de Janeiro State University, Rio de Janeiro, RJ, Brazil. Boulevard 28 de Setembro, 157; Vila Isabel – Rio de Janeiro, RJ, Brazil

## Abstract

Treating skeletal class III malocclusions is one of the biggest challenges in Orthodontics. Given the complexity of these cases, orthognathic surgery is often the best treatment option. However, many patients refuse this treatment due to its risks, morbidity, and costs involved. Alternatively, dental compensation can be planned for some of these skeletal problems. This case report presents a dentoalveolar compensation in the orthodontic treatment of a 20-year-old female patient with class III malocclusion, concave profile, anterior crossbite, mandibular prognathism, maxillary retrusion, and a vertical deficiency in the posterior region. Treatment planning involved a multiloop edgewise archwire (MEAW) associated with intermaxillary elastics with counterclockwise rotation of the occlusal plane in the posterior region of the maxilla aiming at obtaining an increased posterior vertical dimension. After 24 months of treatment, the severe anterior crossbite was corrected, and the skeletal class III relationship was camouflaged. At the end of the orthodontic treatment, it was possible to observe an improved facial profile, a nice smile, and a functional occlusion. The results remained stable at a three-year follow-up. The MEAW, associated with the use of elastics, seems to be an effective treatment option for class III camouflage with reduced posterior vertical dimension with no need for additional anchoring devices but requiring adequate bending of wires and patient compliance.

## 1. Introduction

The diagnosis, treatment, and prognosis of class III malocclusion are often a challenge for orthodontists [1]. The main features of skeletal class III anatomy include mandibular prognathism, maxillary atresia, anterior crossbite, and a reduced skull base angle [2]. However, there are different types of class III malocclusions depending on the predominance of the facial growth (vertical or horizontal) and the mandibular plane angle (open or closed) [3].

Class III malocclusion with a closed mandibular plane angle is characterized by poor vertical growth of the maxillary posterior segment, and, as a consequence, there is a deficiency in the posterior vertical dimension. In such cases, the occlusal plane, in the posterior region, presents a clockwise rotation, with a lack of proportion of the vertical maxillary growth and the mandibular ramus, with an excessive counterclockwise rotation of the mandible. As a consequence, there can be an anterior crossbite with negative overbite [2, 4, 5].

The vertical dimension is believed to be significantly different in skeletal malocclusions, playing an important role in establishing the various sagittal bone relationship morphologies [6]. The primary objective in treating class III malocclusion, with a predominance of horizontal growth, is to restore the skeleton harmony by controlling the occlusal plane and obtaining an appropriate posterior vertical dimension, thus improving the balance between the mandibular ramus height and the posterior maxillary vertical dimension [3, 7].

Multiloop Edgewise Archwire Wires (MEAW), which were introduced by Kim [8, 9], are effective in treating class III malocclusion basically with vertical occlusal plane alteration and distal mass movement of the lower arch. The distal movements of the posterior lower teeth occur through the boot loops and second-order bends (tip-back), along with class III intermaxillary elastics, promoting counterclockwise rotation of the occlusal planes. This improves both the sagittal bone and teeth relationship, allowing a good intercuspal relationship in a short period of time [3].

In the present case, due to the rejection of the patient's surgical treatment and to the lack of vertical harmony between the jaws with a moderate facial aesthetic compromise, counterclockwise rotation of the occlusal plane and a clockwise rotation of the mandible were promoted through orthodontic mechanics and no surgical intervention and without any type of skeletal anchorage. Verticalization and extrusion of the upper and lower molars as well as vestibular proclination of the upper incisors were part of the compensatory treatment of this adult skeletal class III malocclusion with the MEAW technique. The profile and the smile were significantly improved and maintained after a three-year posttreatment follow-up.

## 2. Case Presentation

### 2.1. Diagnosis and Etiology

**Figure 1:**
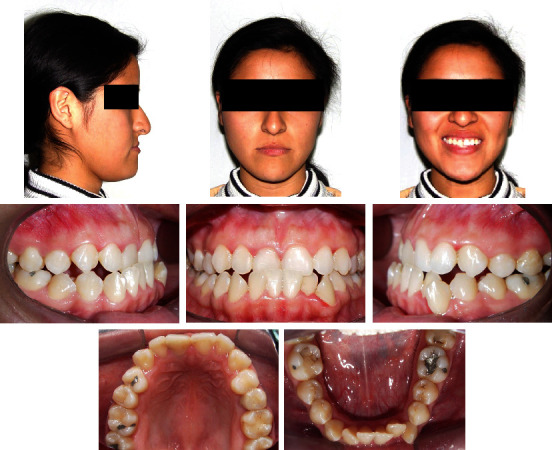
Pretreatment facial and intraoral photographs.

**Figure 2:**
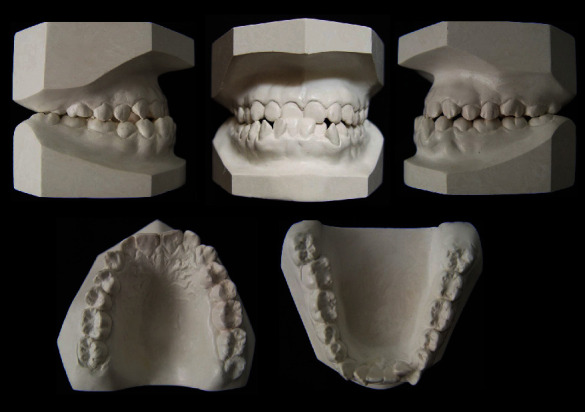
Pretreatment dental casts.

A 20-year-old female was referred to the Orthodontics Department of the School of Dentistry, National University of San Marcos. Her chief complaint was the anterior crossbite, crowded teeth, and a large jaw. She presented a good general state of health and no history of systemic disease. The extraoral frontal examination revealed a mesofacial aspect, a lower prognathic third, and a symmetrical face. The smile showed adequate gingival exposure with upper dental crowding. In the lateral view, a total and concave lower third profile was observed, an apparent lack of development in the sub nasal region, and a protruding lower lip. Intraorally, she had a class III molar and canine relationship, with a negative overjet and overbite of −1 mm and −3 mm, respectively. The space deficiency of the upper arch was -3 mm and -6 mm in the lower arch. The lower midline was 0.5 mm shifted to the left. In the lower arch, the incisors showed a severe lingual inclination and the premolars and molars showed an increased mesial inclination (Figures 1 and 2). No symptoms of temporomandibular joint dysfunction were detected during jaw function and palpation.

**Figure 3:**
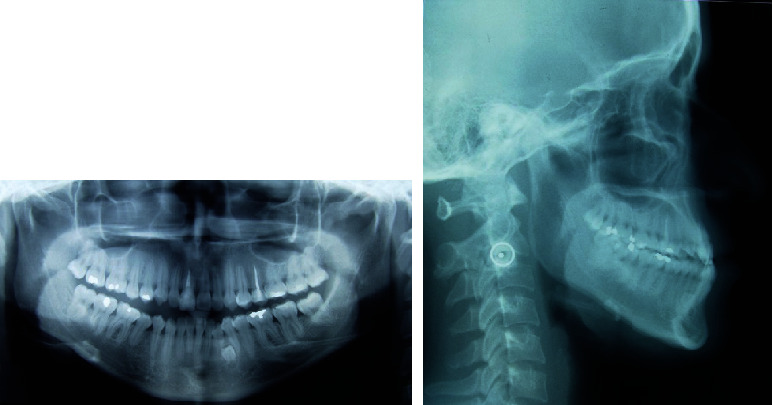
Pretreatment radiographs.

The panoramic X-ray (Figure 3) revealed the presence of the four third molars, and endodontic treatment on the upper right central incisor (11) and the left upper pre-molar (25), with no presence of caries. It was identified the presence of a supernumerary tooth between the left lower canine and the first premolar (33 and 34). The condyles were morphologically asymmetrical. The cephalometric analysis revealed a slight maxillary retrusion (SNA: 79°) and mandible protrusion (SNB: 82°), resulting in a skeletal class III (ANB: −3°; Wits: -11 mm). Facial growth was hypodivergent (FMA: 20.5^0^) and the occlusal plane showed clockwise rotation (Occlusal-SN plane: 22.5°) indicating a deficiency in maxillary posterior vertical dimension.

Cephalometric radiography also showed an increased distal angulation of the maxillary molars and upper and lower incisors retroinclined (1-NA: 15° and 1-NB: 17°) (Figure 3, Table 1).

### 2.2. Treatment Objectives

The overall goals of the treatment plan were to achieve a good skeletal relationship, improve the profile, and establish a stable occlusion. The specific objectives were (1) to correct the sagittal skeletal relationship, (2) promote a counterclockwise rotation of the occlusal plane to incorporate the correct vertical dimension of the maxilla, (3) achieve a clockwise rotation of the jaw to improve facial profile, (4) establish a correct overbite and overjet, (5) establish a class I molar and canine relationship, (6) to eliminate the negative tooth discrepancy, (7) correct the deviation of the lower midline, (8) extraction of the supernumerary tooth, and (9) improve the smile.

### 2.3. Treatment Alternatives

Initially, an orthosurgical treatment was presented for the patient. This would consist of a maxillary Lefort I advancement and a ramus sagittal osteotomy to achieve mandibular clockwise rotation and setback. Due to economic constrains and fear, the surgery was rejected. Therefore, an orthodontic camouflage was considered as an alternative, based on five biomechanics options: (1) teeth extractions (lower first premolars and upper second premolars); (2) intermaxillary Class III elastics; (3) miniplates; (4) extraalveolar miniscrews; and (5) Multiloop Edgewise Archwire (MEAW) technique.

The patient has not accepted extractions apart from third molars nor the use of skeletal anchorage. Thus, the MEAW technique was the chosen option as the occlusal plane could be rigorous controlled. The patient was aware of the need for collaboration regarding the use of intermaxillary elastics and of the need of third molars extractions.

### 2.4. Treatment Progress

**Figure 4:**
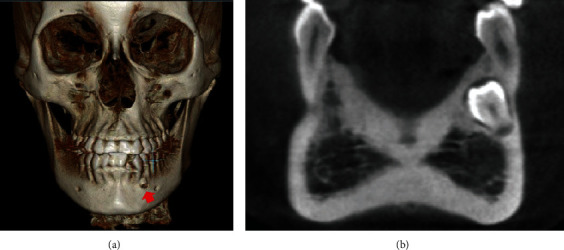
(a) 3D frontal view of the CBCT where the supernumerary tooth is visualized (red arrow) and (b) Coronal section of the supernumerary tooth showing no evidence of contact with the first lower premolar.

**Figure 5:**
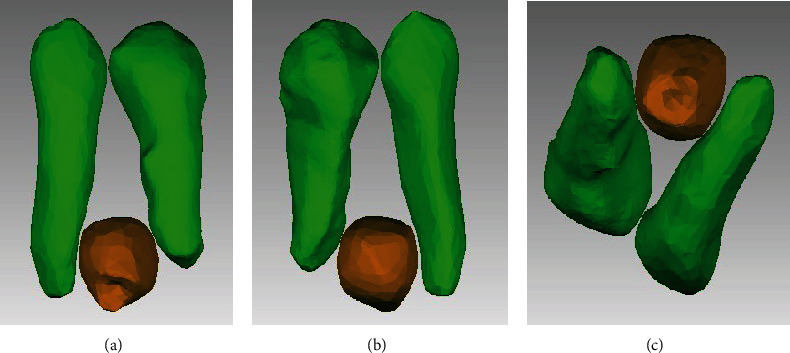
3D reconstruction of supernumerary, cuspid, and premolar teeth: (a) vestibular view of segmented tooth, (b) lingual view, and (c) Inferior view.

**Figure 6:**
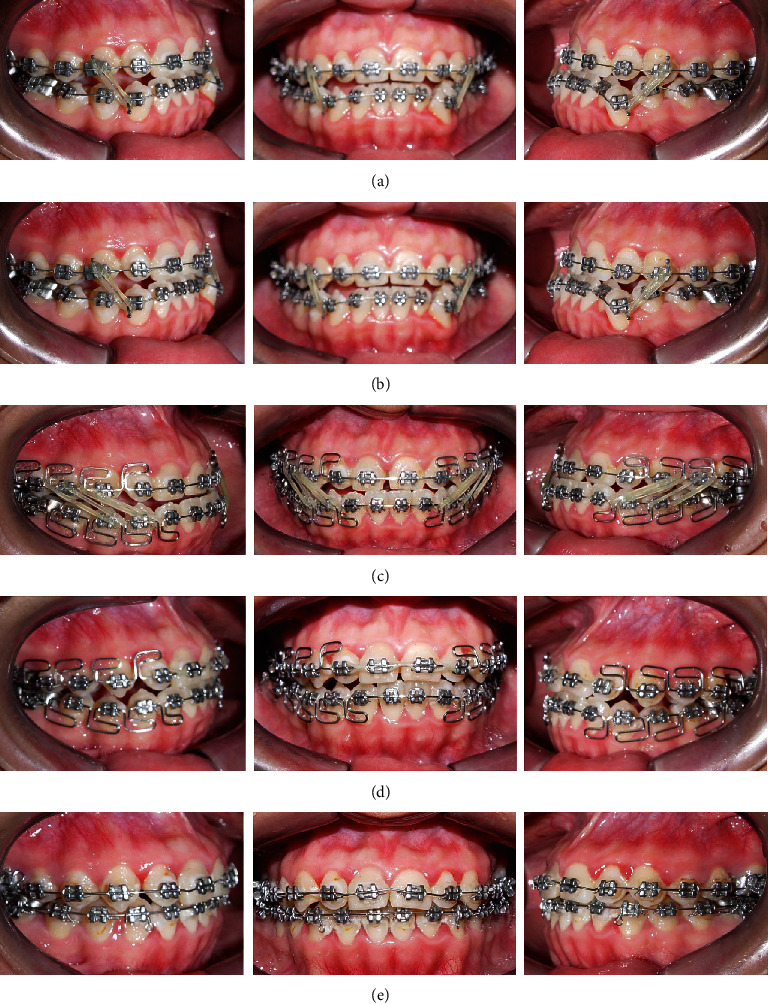
(a) Alignment and leveling with wires 0.014-inch NiTi, (b) MEAW arches with blue elgiloy 0.016 × 0.022-inch, (c) MEAW arches with blue elgiloy 0.016 × 0.022-inch and elastic 3/16 in, 4.5 oz elastic with Class III component, (d) MEAW arches and canine Class I, (e) 0.020-inch Stainless Steel continuous arches with the use of 3/16-in Class III elastics 4.5 oz.

**Figure 7:**
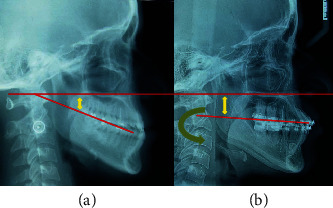
Occlusal plane evaluation, before (a) and at the end (b) of treatment. (a) Deficiency of vertical dimension (short yellow arrow), (b) increase of the vertical dimension (long yellow arrow) with counterclockwise rotation of the occlusal plane (green arrow).

Treatment began with third molar extractions to facilitate distal movements of the upper and lower second and first molars. A cone-beam computed tomography (CBCT) scan was performed to assess the area of the supranumerary tooth; an initial segmentation of the compromised teeth was performed and showed no contact or damages to the adjacent teeth (Figures 4 and 5). The distances between the teeth measured in the tomography were in a range from 0.55 to 1.30 mm. Extraction of the supernumerary tooth was requested but the patient refused the proposal due to unsuccessful previous extraction attempting procedure. Therefore, it was decided to keep the supernumerary tooth as long as annual controlling exams were performed. Full-fixed appliance was placed in both dental arches (0.022 × 0.028-inch slot, 3 M Unitek). First and second molars were banded with prescription tubes MBT 0.022 × 0.028-inch slot (3 M Unitek). Treatment with MEAW arches was divided into five phases, as proposed by Kim [8] and Sato [10, 11]: (1) Alignment and Leveling, where the 0.016-inch, 0.018-inch, and 0.016^”^ × 0.022-inch slot Nickel-Titanium (NiTi) (Unitek-3 M) arches were installed. Short intermaxillary 3/16 in elastic, 2.5 oz of upper canine to lower canine was placed to avoid excessive buccal inclination of the lower incisors. The objectives were to correct rotations, coordinate the upper and lower arches, and facilitate the insertion of the MEAW arch (Figure 6(a)); (2) Elimination of Interferences, which was initially performed by installing an upper MOAW (Modified Offset Arch Wire) arch, made with 0.016 × 0.0022-inch blue elgiloy (RMO) wire. It was used to produce intrusion and buccal inclination of the upper incisors facilitating the crossbite correction (Figure 6(b)). To eliminate dental interference from the posterior segments, upper and lower MEAW arches with 0.016 × 0.022-inch blue elgiloy (RMO) wires were installed. Step-up bends for the upper and lower molars associated with tip-back bends for molar and lower premolar verticalization and class III (3/16-in, 6.5 oz) intermaxillary elastics were incorporated. (Figure 6(c)); (3) Mandibular displacement, with downward and backward rotation, helped achieving an adequate molar and canine Class I relationship, as well as correcting the overbite and overjet (Figure 6(c)); (4) Reconstruction of the Occlusal Plane, modification of the posterior occlusal plane by incorporating a step down of the upper molars and a tip-back of the lower molars. In this phase, the occlusal plane was rotated (counterclockwise) increasing the vertical dimension of the maxilla (Figure 6(d) and 7(b)); and (5) Obtaining a Physiological Occlusion, with the use of a 0.020-inch stainless steel (SS) continuous arch and class III intermaxillary elastics, to achieve a good intercuspation (Figure 6(e)).

After 15 months of MEAW mechanics, the treatment continued with a 0.020-inch and 0.018 × 0.025-inch rectangular stainless steel arches and Class III elastics (3/16 in, 4.5 oz) to extrude the upper molars and continue increasing the posterior occlusal dimension. Treatment was completed within 24 months. A Hawley removable appliance associated to bonded canine to canine lower lingual wire were used as retainers (0.7 mm SS), supervised from 3 to 3 months.

### 2.5. Treatment Results

**Figure 8:**
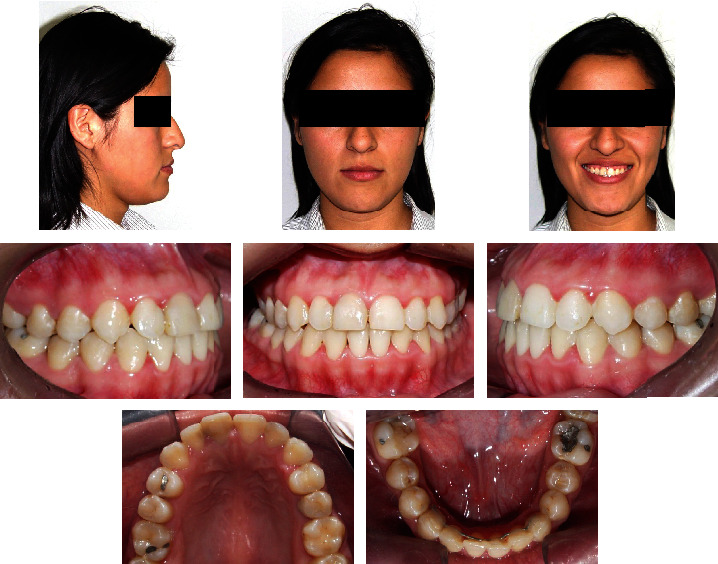
Posttreatment extraoral and intraoral photographs.

**Figure 9:**
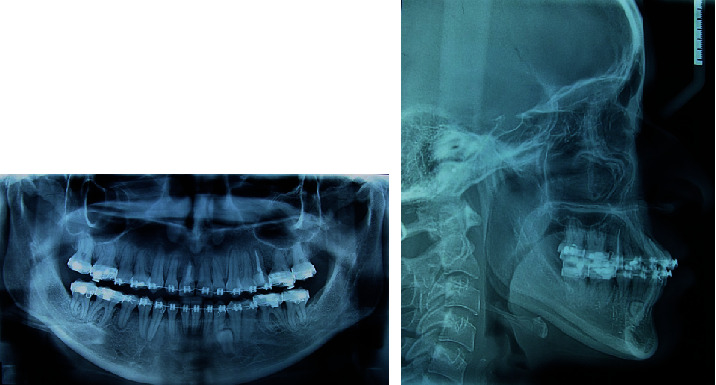
Posttreatment radiographs.

**Figure 10:**
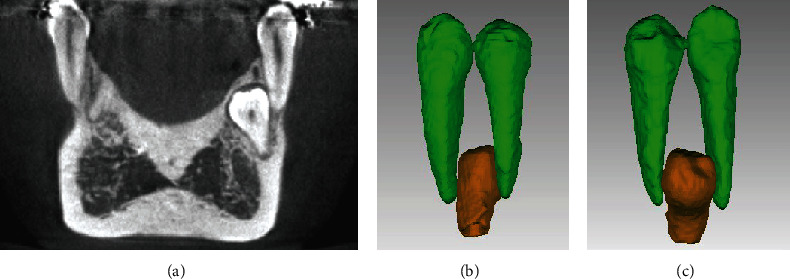
Final CBCT. (a) Axial view of the supernumerary tooth; 3D reconstruction of teeth: (b) vestibular view of segmented teeth, (c) lingual view.

The final registries showed that the majority of treatment targets were accomplished. Extraoral photographs indicated significant improvement of the facial profile and lip position. The lack of development in the subnasal region, and the protrusion of the lower lip were camouflaged. The smile became more aesthetic, and the patient reported great satisfaction with the results. Intraorally, the anterior crossbite was corrected, a Class I Canine and a Class III molar functional relationship was achieved, with adequate overjet and overbite. The dental arches were aligned and leveled and the upper and lower dental midlines were coincident (Figure 8). Panoramic X-ray showed a verticalization of the upper and lower molars and premolars, with acceptable parallelism and no signs of significant bone or root resorption (Figure 9). In order to reevaluate the position of the remaining, a CBCT was required and the area segmented using ITK-SNAP 3.8 (USA) (Figure 10). There was no contact nor damages between the extra tooth and neighbouring teeth. Final Cephalometric radiography—taken immediately before appliance removal—(Figure 9, Table 1) revealed an improved skeletal relationship (ANB: 1°, SNA: 81.5°, SNB: 80.5°, Wits: -5 mm). Regarding the dentoalveolar changes, it is possible to observe the protrusion of the upper and lower incisors (1-NA: 34°, 1-NB: 23° and IMPA: 83°) and a decrease in the inclination of the occlusal plane (Occ. Plane-SN: 14°), which reflected an increase in the vertical dimension of the maxilla (Figure 7). Cephalometric superposition revealed the improvement of the facial profile with a slight increase in facial height (as shown in Table 1: FMA: 23°).

**Figure 11:**
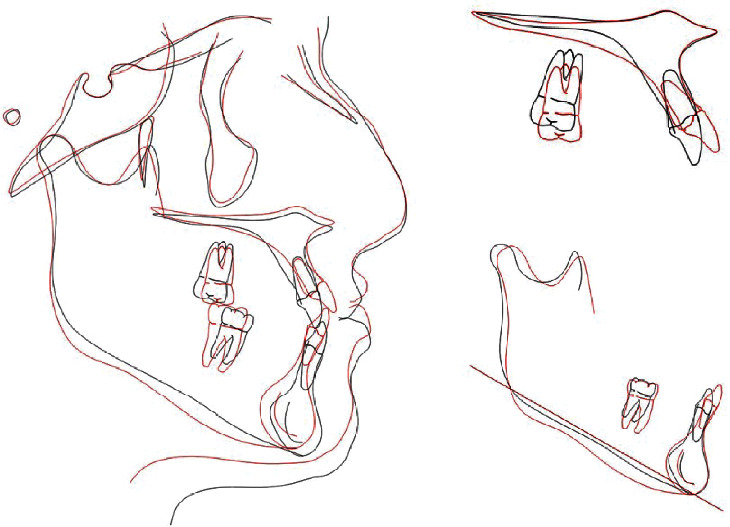
Pretreatment (black line) and posttreatment (red line).

**Figure 12:**
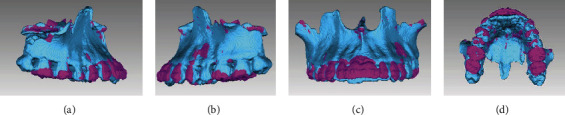
Initial (blue color) and posttreatment (pink color). (a) Right side, (b) left side, (c) frontal view, and (d) occlusal.

**Figure 13:**
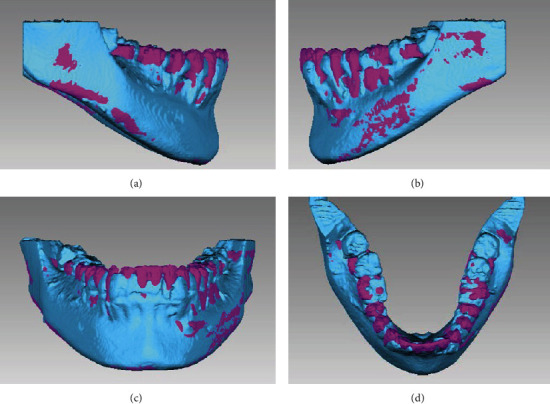
Initial (blue color) and posttreatment (pink color). (a) Right side, (b) left side, (c) frontal view, and (d) occlusal.

The upper and lower molars were uprighted and the upper extruded as well resulting in an anticlockwise rotation of the occlusal plane (OccPlane-SN: 14°). The vestibuloversion of the upper and lowers incisors can be observed (Figure 11). Three-dimensional (3D) superposition of the jaws was performed using Geomagic Qualify 2013 (USA), highlighting the extrusion of the upper molars and the vestibuloversion of the upper incisors (Figure 12). In the 3D superposition of the mandible, no extrusion of the lower molars and mild vestibuloversion of the lower incisors was observed (Figure 13).

**Figure 14:**
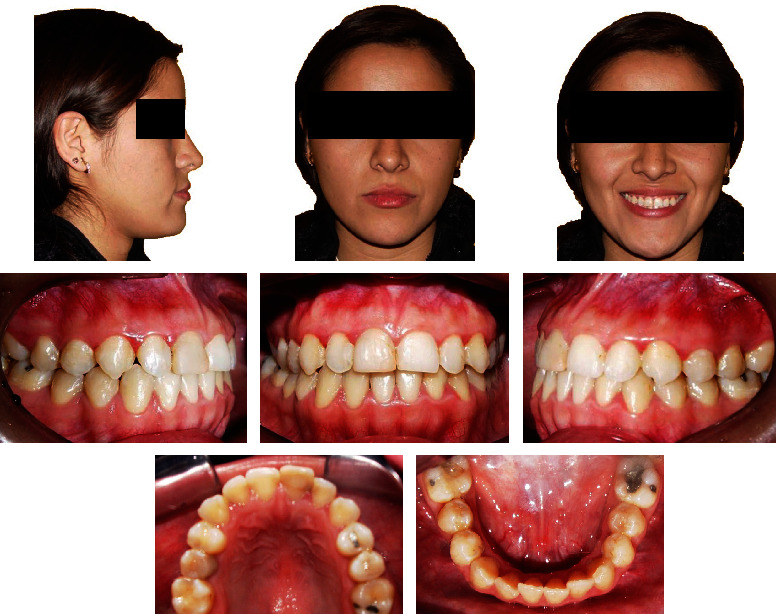
Intraoral and extraoral photographs at 3 years posttreatment.

After three years of posttreatment, the position of the lips was maintained and profile remained straight. The patient underwent nasal plastic surgery, with a significant improvement of the facial profile. Functional molar Class III and canine Class I were maintained, although the patient lost the lower retention after24 months of control. A slight relapse was observed in the lower incisors; however, an occlusion with acceptable stability was observed (Figure 14).

## 3. Discussion

For correct diagnosis and orthodontic treatment plan, it is important to evaluate the morphological characteristics of the different vertical and sagittal skeletal alterations types, observed in Class III malocclusion, dolichofacial, mesofacial, or brachyfacial, each one with its own characteristics [2–4]. A close relationship has also been identified between the vertical behavior of the occlusal plane and the establishment of a certain sagittal skeletal alteration [6]. Thus, there are certain types of Class III malocclusions with an imbalance between the vertical growth of the mandibular ramus and the vertical growth of the maxilla in the posterior region, which is hypodeveloped causing a deficiency of vertical descent of the occlusal plane. As a consequence, the lower jaw loses the adequate vertical occlusal support and moves forward [3]. This morphological feature is present in our patient. The deficiency of the occlusal support and in the vertical dimension can be identified in the facial and dentoalveolar analysis and mainly, in radiographic evaluation (Figure 7 and Table 1). The patient reported that she had already consulted other specialists, and that all had proposed orthognathic surgery as the only treatment option for her case. Traditionally, this type of treatment has been the most effective way to correct skeletal discrepancies. The first treatment choice for this patient was orthognatic surgery. However, the patient refused the surgical intervention. Therefore, a compensatory orthodontic treatment was considered and the options were the use of class III intermaxillary elastics, treatment with miniplates [12] or extraalveolar mini implants [13, 14], treatment with premolar extractions [10], and the adoption of the MEAW [7, 8] technique.

Since the patient had an FMA of 20.50°, a treatment with premolars extractions could reduce even more the occlusal support in the posterior region. The maintenance or increase of the occlusal support is fundamental to prevent the clockwise mandibular rotation and also to improve the facial profile. The use of Class III intermaxillary elastics alone would cause a counterclockwise rotation of the occlusal plane by increasing the posterior vertical dimension, but would not be sufficient to upright the lower molars and premolars as well as to perform a thorough control of the occlusal plane. The use of miniplates would involve minor surgical procedures, also rejected by the patient. The use of extraalveolar mini implants may provide adequate anchorage for the verticalization of the premolars and lower molars, along with a counterclockwise rotation of the occlusal plane, but with minimal extrusion and verticalization of the upper molars, which is required to increase the vertical dimension. In addition, this technique seems to be effective for Dolichofacial Class III patients [13]. Seeking to obtain counterclockwise rotation of the occlusal plane and a clockwise rotation of the mandibular plane, we opted for the MEAW technique to correct the vertical imbalance between the mandibular and the maxillary arches [3]. This treatment modality was developed by Kim [15–18] and is considered effective for class III corrections, severe open bite, lateral deviation of the mandible, and TMJ problems. The technique does not require skeletal anchoring devices; however, the loops require manual operator skills, and patient collaboration is of utmost importance.

The tip-back, step-up, and/or step-down bends incorporated into the arches, associated to intermaxillary elastics, generate significant vertical dental displacement, allowing the occlusal planes to be reconstructed effectively through moving the posterior teeth vertically [19–22]. The presence of the loops provides versatility and flexibility to the MEAW technique; therefore, the forces produced are more physiological than the ones generated by the continuous arches [9]. This alveolar compensation process takes place in a relatively short period of time due to the simultaneous movement that occurs in all teeth.

The treatment performed consists of several phases, beginning with the alignment of the dental arches with light and continuous wires seeking to prepare it for a more passive insertion of the MEAW arch (Alignment and leveling phase). Then, the anterior crossbite was corrected with the MOAW (modified offset archwire) arch and all types of occlusal contact of the molars and second premolars were eliminated, with posterior intrusion. This gave the mandible more freedom for a sagittal movement (Interference elimination phase). Once the occlusal interference was eliminated, the jaw was relocated in the posterior direction (clockwise rotation) and the adequate vertical dimension is obtained, with occlusal contacts just at the level of the first premolars. At this point, a class I canine relationship and a functional class III molar relationship were achieved. The facial profile was favorably modified (mandibular replacement phase). The relocated mandible was stabilized so that intercuspidation of all molars and second premolars was sought, now with extrusive movements, mainly of the upper molars (Occlusal plane reconstruction phase). Thus, the stability of the jaw was achieved and an acceptable canine and molar relationship were sought to increase occlusion throughout the arch (phase of obtaining a physiological occlusion) [5]. All phases required the use of 3/16 in, 6.5 oz elastics, which were installed from the second upper loop to the first lower loop. When using double elastics per side, the elastic force should be reduced (4.5 oz) (Figure 6(c)).

Rotation of the occlusal plane vertically in the treatment of Class III malocclusion has been reported by several authors. From studies using modern noninvasive tools such as finite element analysis proposed by Roberts et al. [13], who explained that the occlusal plane could rotate counterclockwise through a statically determined biomechanical system and without the need for premolar extractions or orthognathic surgeries. Clinical studies such as the one carried out by He et al. [23] who, in a total sample of 44 patients without growth, determined that the MEAW arch and the Class III elastics were an appropriate strategy for the management of the occlusal plane in the treatment of Class III malocclusion, even in hyperdivergent patients. The opposite rotation between the occlusal plane and the mandibular plane to return the vertical dimension to a patient with hypodivergent Class III was also highlighted by Park and Bullen [11]. The result of vertical occlusal plane management in our patients was consistent with these previous studies.

During the camouflage treatment of the Class III malocclusion, the MEAW arch can also be used in the early stages and can then be replaced by mini implants and continuous arches [24]. In the present case, it was decided to maintain the MEAW arch until the final stages of the treatment, as it was considered to have a stricter three-dimensional control of torque, tipping, extrusion, and intrusion of tooth movement.

The MEAW arch can be made of 0.017 × 0.025-inch stainless steel archwire or with 0.016 × 0.022-inch “blue elgiloy” wire. In this treatment phase, second-order bends (tip-back, tip-forward, step-up, and step-down) are important for the establishment of the modified occlusal plane. Third-order bends (torque) and arch coordination should be considered during all treatment phases to avoid edge-to-edge intercuspidation of teeth [5, 13]. In our case, the relation between upper and lower right second molars was not ideal, finalizing almost in a tip-to-tip relation. The molar uprighting procedure of the molars could have caused this condition. However, it did not create any occlusal interference and the patient refused to elongate treatment to correct it. Cephalometric superimposition reveals the verticalization of the upper and lower posterior teeth, as well as the extrusion of the upper posterior teeth, enabling the counterclockwise rotation of the occlusal plane (OccPlane-SN: 14°) and the incorporation of the vertical dimension. There was also a slight clockwise rotation of the mandibular plane (FMA: 23°) and improvement of the patient's facial profile. Although there was greater proclination of the upper incisors (1-NA: 34°) than of the lower incisors (1-NB: 23°), there was no decrease in dental exposure during smile.

Thanks to the current technological management of tomographic information [25, 26], 3D superimposition of the maxilla and mandible was performed. Extrusion of the upper molars and the proclination of the upper incisors are clearly observed (Figure 12), confirming the two-dimensional results provided by the cephalometric overlays. In the 3D superimposition of the lower jaw (Figure 13), almost none extrusion and the slight verticalization of the lower molars are corroborated, as well as the slight proclination of the lower incisors. We can then suggest that the upper and posterior dentoalveolar extrusion was decisive in obtaining the balance between the mandibular ramus and maxillary height as described by previous authors [3, 4, 6, 16].

After extracting the 4 third molars, the patient was fearful of having the supernumerary extracted. The supernumerary tooth had no contact with the neighboring teeth, which was corroborated by the initial 3D segmentation performed on the compromised teeth (Figures 4 and 5). The distance between the supernumerary tooth and the canine and the premolar measured in the tomography were in a range from 0.55 to 1.30 mm, thus it was decided to follow it up during the orthodontic treatment and along periodic control appointments. The patient was alerted of the possible consequences of keeping it and of future extraction need in case the tooth contacted the neighbor teeth during the application of the mechanics or in case of any pathological development, as indicated by previous studies [27]. At the end of the treatment, a CBCT evaluation and segmentation of the supernumerary and its neighboring teeth was performed, corroborating the normal morphology of the teeth and the lack of contact with adjacent teeth (33 and 34) (Figure 10). The patient showed no discomfort in the region during treatment phases.

The MEAW technique was found effective for compensatory treatment of Class III malocclusion [28, 29], although mild discomfort was manifested by the patient at the beginning of treatment due to the presence of the bends and the use of elastics. It is critical that good quality arches are used to support the additional bends to be incorporated. These bends (tip-back, step-up, step-down, tip-forward, and torque) are incorporated into the MEAW arches in each phase of the treatment and the arches made with blue elgiloy wire should be subjected to heat treatment (470°C for approximately 3 minutes). It is vital that the patient be aware of the need to use the intermaxillary elastics since, without them, the MEAW technique will not be effective [5, 8].

Following the San Marcos University protocol, the final radiographs are registered with the brackets in position, in case there is a need to perform any additional dental movement. Three years after the end of treatment, the patient showed an adequate profile, a pleasant smile, and no sign or symptoms of temporomandibular dysfunction. The patient was followed up regularly for 2 years after treatment. Due to moving away, she did not show up during the third year. The use of retention was discontinued and she returned presenting a mild relapse of the lower incisors with a few rotations but, in general, the occlusion showed acceptable stability as described by previous authors who used the MEAW arches [19].

This case shows that, although the limitations imposed by the patient in not accepting surgery nor skeletal anchorage, it was possible to manage her chief complaint and to obtain good results through the knowledge of mechanical concepts and hand skills improving the aesthetics and occlusal conditions.

## 4. Conclusion

A severe skeletal Class III with a deficiency of posterior vertical dimension in the maxilla had an alternative treatment with the MEAW technique. The objectives were to obtain a counterclockwise rotation of the occlusal plane by extruding the upper molars and verticalizing the lower teeth, creating an acceptable occlusion along with a clockwise rotation of the lower jaw. Thus, the lower facial third and the patient's overall profile were improved. This MEAW technique requires some bending skills and orthodontic training, as well as an adequate compliance of the patient in the use of the intermaxillary elastics, but the results that can be obtained, in some cases, justify the attempt to overcome these difficulties.

## Figures and Tables

**Table 1:** Pretreatment and posttreatment cephalometric values.

Measurement	Norm	Pretreatment	Posttreatment
SNA (^o^)	82	79	81.5
SNB (^o^)	80	82	80.5
ANB (^o^)	2	-3	1
Wits	-1	-11	-5
Convexity (^o^)	0	-4.5	1
*y*-axis (^o^)	59.9	50.5	51.5
Facial angle (FH-NPo) (^o^)	87.8	102	100
Mandibular plane to SN (SN-GoGn) (^o^)	32	38.5	38
FMA (^o^)	25	20.5	23
IMPA (^o^)	90	75.5	83
Occlusal plane to SN (OP-SN) (^o^)	14	22.5	14
Maxillary incisor-NA (U1-NA) (^o^)	22	15	34
Maxillary incisor-NA (U1-NA) (mm)	4	11	22
Mandibular incisor-NB (L1-NB) (^o^)	25	17	23
Mandibular incisor-NB (L1-NB) (mm)	4	11.5	20
1–1 (^o^)	130	150	121
LS-S (mm)	0	-11	-5
LI-S (mm)	0	4	4
